# Cortical Auditory Evoked Potentials in Children with a Hearing Loss: A Pilot Study

**DOI:** 10.1155/2012/250254

**Published:** 2012-01-12

**Authors:** Amineh Koravand, Benoît Jutras, Maryse Lassonde

**Affiliations:** ^1^École d'Orthophonie et d'Audiologie, Université de Montréal, C.P. 6125, Succursale Centre-Ville, Montréal, Qc, Canada H3C 3J7; ^2^Centre de Recherche du CHU Sainte-Justine, 3175 Côte Sainte-Catherine, Montréal, Qc, Canada H3T 1C5; ^3^Centre de Recherche en Neuropsychologie et Cognition, Université de Montréal, C.P. 6125, Succursale Centre-Ville, Montréal, Qc, Canada H3C 3J7

## Abstract

*Objective*. This study examined the patterns of neural activity in the central auditory system in children with hearing loss. *Methods*. Cortical potentials and mismatch responses (MMRs) were recorded from ten children aged between 9 and 10 years: five with hearing loss and five with normal hearing in passive oddball paradigms using verbal and nonverbal stimuli. *Results*. Results indicate a trend toward larger P1 amplitude, a significant reduction in amplitude, and latency of N2 in children with hearing loss compared to control. No significant group differences were observed for the majority of the MMRs conditions. *Conclusions*. Data suggest that the reduced auditory input affects the pattern of cortical-auditory-evoked potentials in children with a mild to moderately severe hearing loss. Results suggest maturational delays and/or deficits in central auditory processing in children with hearing loss, as indicated by the neurophysiological markers P1 and N2. In contrast, negative MMR data suggest that the amplification provided by the hearing aids could have allowed children with hearing loss to develop adequate discriminative abilities.

## 1. Introduction

Sensory hearing loss often affects speech perception due to a decreased audibility of the signal as well as decreased temporal analysis ability [1–3]. Studies have demonstrated the influence of hearing loss on auditory temporal ordering, a task which involves the central auditory system [4–6]. The lower performance of children with hearing loss in this task could be caused by central auditory neurophysiological deficits.

Auditory neurophysiological functions have been measured in adults and children with hearing loss [7–11]. Sensory hearing loss in adults induced a delay in the latency of N1, N2, and a reduction in N2-P2 amplitude [8]. Oates et al. [7] investigated the N1, N2, MMN, and P3, presented at 65 and 80 dB SPL, and found a latency prolongation and an amplitude reduction of these components in adults with hearing loss compared to those of the control group at both levels of presentation. However, an earlier study did not reveal any significant differences in the latencies of N1, P2, and P3 components between adults with hearing loss and their normal-hearing controls [11]. Several factors could account for these differential findings, such as participants' age, age at onset of hearing loss, type and/or degree of hearing loss, level of stimulus presentation, and type of stimuli used.

In children, latency changes in the cortical-auditory-evoked potentials (CAEPs) have been used to document auditory system plasticity and recovery from auditory deprivation following cochlear implantation [10, 12]. Congenitally deaf children who are fitted with cochlear implants during a sensitive period of early childhood show normal central auditory maturation within six months of implant use as demonstrated by changes in P1 latency [10, 12]. Interestingly, in children with sensory hearing loss and those with auditory neuropathy, the P1, N1, and P2 components are present only in those children exhibiting good speech perception skills [9]. However, the children with and without good speech perception skills were not age-matched and this factor could have influenced the results, since CAEPs show substantial changes with maturation [13, 14].

To determine CAEPs potential clinical benefits in assessing central auditory functions in children with hearing loss, a clear understanding of the effects of a sensory hearing loss on CAEPs is needed. The main objective of the present study is to explore central auditory neurophysiological functions in children with hearing loss wearing hearing aids. If CAEPs are affected by sensory hearing loss, cortical auditory measures could become neurophysiological markers in clinical audiology for evaluating young children for whom central auditory functions are difficult to assess behaviourally.

## 2. Materials and Methods

### 2.1. Participants

Two groups of nine-to-ten years old female children participated in the study: five with sensory hearing loss (mean age: 9 : 10 years, SD = ±3 mo) and five with normal hearing (mean age: 9 : 11 years, SD = ±3 mo). Participants with normal hearing had auditory detection threshold at 15 dB HL or less between 500 Hz and 8 kHz, bilaterally (re: ANSI, 1996 [15]). Average hearing sensitivity thresholds of children with hearing loss, based on the average of 500, 1000, and 2000 Hz thresholds, were within the limits of mild to moderately severe hearing loss (according to Clark (1981) classifications [16]). All participants were right handed, as measured by an adapted protocol to assess laterality dominance [17]. The study was approved by the Ethics Committee of the Sainte-Justine Hospital.

### 2.2. Stimuli

Three pairs of synthetic stimuli were used for this study: one verbal and two nonverbal pairs. The verbal stimuli consisted of two syllables: /ba/ and /da/. These stimuli were selected from the CD-ROM, Speech Production, and Perception I [18]. The first pair of nonverbal stimuli consisted of synthesized transformations of /ba/-/da/, generated using two softwares, Dr. Speech and Mitsyn [19, 20]. Only the second and third formants were used to create the nonverbal stimuli (for more detail on the syllable transformation, see Mody et al. [21]). Using only these two formants, the stimuli are recognized as nonverbal sounds [21]. The second pair of nonverbal stimuli was the pair of a 1 kHz pure tone and a wide-band noise. Stimuli were 250 ms in duration with 2.2 ms rise and fall times.

The stimuli were presented with a computer *(DELL)* using Stimaudio (NeuroScan Inc.) and the *Stim^2^* software. They were presented to the right ear through insert-earphone (E-A-RTONE 3A), connected to an audiometer (Interacoustics, AD229b model), at 70 dB HL for children with normal hearing and between 85 and 105 dB HL for those with hearing loss (See Table 1). Stimuli were presented in a passive oddball paradigm, with standard stimuli (syllable /ba/, nonverbal /ba/ and a 1 kHz pure tone) of 85% probability of occurrence and deviant stimuli (syllable /da/, nonverbal /da/ and wide-band noise) with a 15% probability of occurrence. The interstimulus interval (ISI) was one second. The order of stimulus presentation was pseudorandomized within a run, with no two deviants occurring in succession and no run beginning with a deviant stimulus. Any deviant stimulus was always preceded by at least three standard stimuli. A thousand counterbalanced trials for each pair of stimuli were recorded.

### 2.3. Electrophysiological Recordings

The cortical responses were digitally recorded using a high-density system, Scan 4.0 software (NeuroScan, Inc., USA), with SynAmps amplifiers and from 128 Ag/AgCl electrodes. Electrophysiological signals were acquired at a sampling rate of 250 Hz, with an analog online bandpass filtering from 0.1 to 100 Hz using the SynAmps amplifiers running on a* Dell* computer. An electrode located on the forehead (Fpz) served as ground and reference was located at the vertex. Electrode impedance was kept under 7 kΩ for mastoid, central, and frontal regions and below 15 kΩ for ocular and peripheral regions.

### 2.4. Procedure

The children were seated in a chair in a double-walled sound-proof booth. Participants watched a movie or cartoon of their choice on a computer monitor with the sound off. They were told to ignore the auditory input and to focus their attention on the movie. Total testing duration was approximately between 90 and 120 minutes.

### 2.5. Data Analysis

Using BrainVision Analyser program on an IBM computer, the data were corrected for eye movements using Gratton and Coles algorithm [22]. They were next digitally filtered using a filter of 1–15 Hz at 24 dB/octave. These data were rereferenced to both mastoids electrodes. Eye movements and epochs with other artefacts were rejected based on voltage criteria (±100 *μ*V). The timeframe of analysis was from −100 ms to 700 ms. Data were baseline corrected to −50 ms. Auditory cortical components were defined as followed: P1 and N1 were the first positive and negative waveforms in the time window of 50–100 ms and 80–120 ms, respectively. They are followed by a positive peak, defined as P2 within the time window of 100–160 ms, and N2, the second negative peak at 200–280 ms. Amplitude values were measured from baseline to peak for each component, and latency values were measured relative to the onset of stimulus presentation.

MMRs were computed according to the following procedure: ERPs evoked by a standard stimulus were subtracted from ERPs evoked by the presentation of a deviant stimulus for each participant. Responses to standard stimuli that immediately followed the presentation of deviant stimuli were excluded from the standard stimulus average. Two MMRs were observed with the pair 1 kHz pure tone wide-band noise; a first negative peak was measured from 115 to 200 ms and a positive slope was observed from 200 to 330 ms. However, only one prevalent negative response from 115 to 260 ms was observed with the nonverbal and verbal pairs. For each participant, the latency of the most negative or positive peak was measured for the MMRs by using a peak amplitude automatic detection.

## 3. Results

### 3.1. CAEP Components

**Figure 1:**
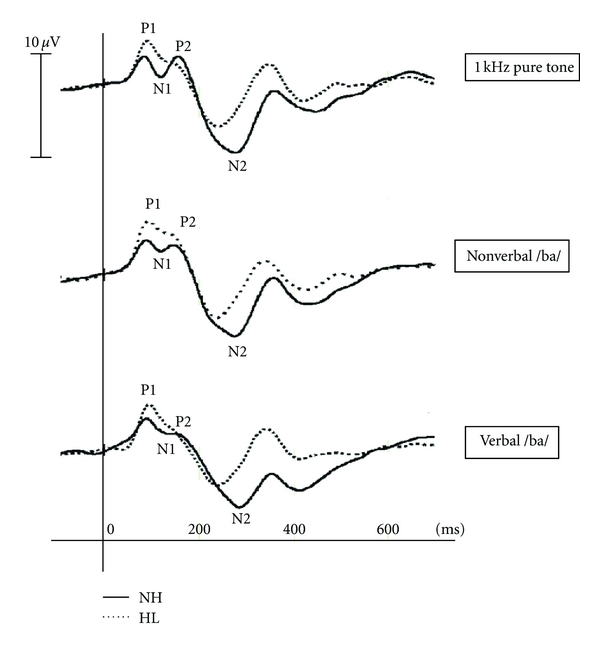
Waveforms recorded at FCz electrode from five children with normal hearing—NH (solid line) and five with hearing loss—HL (dashed line) with 1 kHz pure tone (top), nonverbal /ba/ (middle) and /ba/ (bottom) stimuli.

Statistical analyses were conducted on the amplitude and latency values of the standard sound waveforms because they were better defined and had a clearer morphology compared to those obtained from deviant waveforms. P1, N1, P2, and N2 were observed clearly in children with normal hearing with the three stimuli (Figure 1). By contrast, the N1 and P2 components were not well defined in some children with hearing loss. Therefore, only the P1 and N2 components, which were clearly identified in all participants, were analyzed.

### 3.2. P1 and N2 Latency and Amplitude

**Figure 2:**
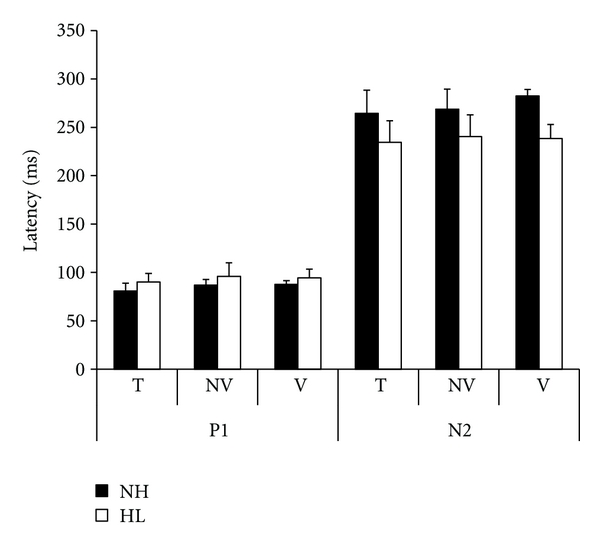
P1 and N2 mean latency values and standard deviation recorded of five children with hearing loss (HL) and five children with normal hearing (NH) with 1 kHz pure tone (T), nonverbal /ba/ (NV), and verbal /ba/ (V) stimuli.

**Figure 3:**
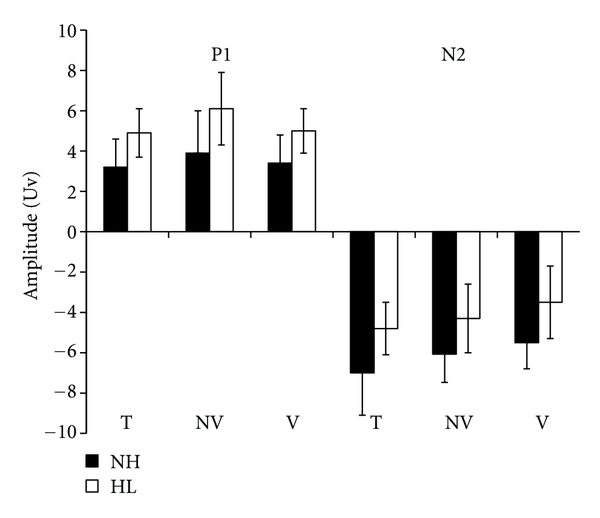
P1 and N2 mean amplitude values and standard deviation of five children with hearing loss (HL) and five children with normal hearing (NH) with 1 kHz pure tone (T), nonverbal /ba/ (NV), and verbal /ba/ (V) stimuli.

Using SPSS software, a two-way ANOVA was performed (Group, Stimulus type) with repeated measures on the second factor, for both P1 and N2 latency and amplitude measures (Figures 2 and 3).

#### 3.2.1. Latency

With regard to P1 latency, results revealed a significant effect for the main Type factor only (verbal /ba/, nonverbal /ba/ and 1 kHz pure tone) [*F*(2,16) = 7.85, *P* < .01]. *t*-tests were conducted, applying Bonferroni corrections to adjust for multiple comparisons (*P* < .016). Results revealed only a significant latency prolongation for the verbal /ba/ than the 1 kHz pure tone [*t*(9) = 3.2, *P* < .016] and for the nonverbal /ba/ than 1 kHz pure tone [*t*(9) = 3.95, *P* < .016].

As pertains to N2 latency, a significant latency reduction was observed in children with hearing loss comparatively to the latency value of children with normal hearing [*F*(1,8) = 9.01, *P* < .01]. Results revealed a significant effect for the Type factor too [*F*(2,16) = 3.9, *P* < .05]. However, no significant difference was observed between the three types of stimuli when *t*-tests with Bonferroni corrections (*P* < .016) were applied.

#### 3.2.2. Amplitude

Regarding P1 amplitude, results revealed a significant effect for the Type factor only [*F*(2,16) = 5.5, *P* < .01]. The main Group factor failed to reach significance but a trend was observed [*F*(1,8) = 4.03, *P* = .08]. For the significant Type factor, a *t*-tests, with Bonferroni corrections (*P* < .016), revealed a significant greater amplitude for the 1 kHz pure tone than the nonverbal /ba/ [*t*(9) = 3, *P* < .016] only.

For the N2 amplitude, a significant amplitude reduction was observed in children with hearing loss comparatively to the amplitude value of children with normal hearing [*F*(1,8) = 5.8, *P* < .05]. Results also showed a significant effect for the type factor [*F*(2,16) = 3.8, *P* < .05]. *t*-tests with Bonferroni corrections (*P* < .016) demonstrated a significant greater amplitude for the 1 kHz pure tone than the verbal /ba/ [*t*(9) = 3.1, *P* < .016] only.

### 3.3. Mismatch Responses (MMRs)

**Figure 4:**
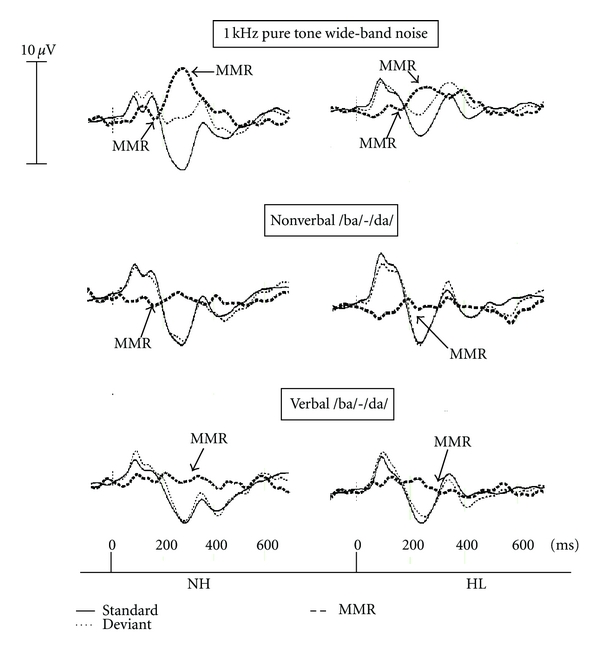
The grand average ERPs of five children with normal hearing (NH) and the five children with hearing loss (HL), elicited by the standard stimuli (solid lines): 1 kHz pure tone (top), nonverbal /ba/ (middle), and /ba/ (bottom); by the deviant stimuli (dotted lines): wide-band noise (top), nonverbal /da/ (middle), and /da/ (bottom). The mismatch response (MMR) is represented by a bold dashed line.

A two-way ANOVA was performed (Group, Stimulus type) with repeated measures on the second factor, for both the MMR latency and amplitude measures (Figure 4).

#### 3.3.1. Latency

With regard to negative MMR latency, results revealed a significant effect only for the main Type factor (verbal /ba/-/da/ pair, nonverbal /ba-/da/ pair and 1 kHz pure tone and wide-band noise pair) [*F*(2,16) = 23.3, *P* < .001]. A *t*-test with Bonferroni corrections (*P* < .016) demonstrated a significant latency prolongation for the verbal /ba/-/da/ pair as compared with the 1 kHz pure tone and wide-band noise pair [*t*(9) = 6.9, *P* < .016] and also comparatively to the nonverbal /ba/-/da/ pairs [*t*(9) = 4.3, *P* < .016].

#### 3.3.2. Amplitude

Regarding the negative MMR amplitude, results revealed no significant effect for the two main factors nor for the two-way interaction Group × Type. A positive MMR component was observed with the pair of 1 kHz pure tone and wide-band noise (Figure 4). A *t*-test was conducted on the amplitude and latency values. Results revealed a significant difference between the two groups for the amplitude value [*t*(8) = 1.8, *P* < .05], but not for the latency value [*t*(8) = .53, *P* = .23].

## 4. Discussion

The aim of the present research was to study the patterns of the neurophysiological activity in the central auditory system in children with hearing loss as compared with children with normal hearing. Differential findings were observed with regard to the principal cortical components and the MMR results.

### 4.1. Cortical Principal Components

P1 amplitude tended to be greater, N1 and P2 components less defined, and amplitude and latency of N2 reduced in children with hearing loss compared with the results of the children with normal hearing. These findings will be discussed according to three factors: the presentation level, the maturation of the central auditory system, and the deficit in the central auditory system.

### 4.2. Presentation Level

The stimuli were presented between 80 and 105 dB HL for the children with hearing loss and at 70 dB HL for the children with normal hearing. The higher level of stimulus presentation (in dB HL) could have contributed to the large amplitude of P1 and to the shorter latency of N2. Oates et al. (2002) found that the amplitude of the N1 and the P300 was larger and their latency shorter at 80 dB SPL compared to 65 dB SPL in adults with hearing loss [7]. In normal-hearing adults, as the intensity increases, peak latencies of P1, N1, P2, and N2 decrease and their peak amplitudes increase [23].

However, the results of the present study were partially in agreement with those results. There were only two indications that the level of presentation could modulate waveform characteristics. In fact, P1 amplitude was larger and N2 latency was shorter in children with hearing loss comparatively to children with normal hearing. The findings indicate that the level of presentation could affect differently the two components.

### 4.3. Maturation of the Central Auditory System

The P1 waveform changes in a complex manner in children. P1 decreases systematically in latency and/or amplitude to reach adult values almost at the age of 14-15 years [24] or 20 years [25]. The maturation of CAEPs has been investigated in children who received their cochlear implant between 18 months and six years of age, with the average age of implantation being 4.5 years [26]. The CAEPs, and in particular, the peak latency of P1, appeared to mature at the same rate as in children with normal hearing but were approximately delayed by the corresponding length of auditory deprivation [26]. This finding emphasizes that once adequate auditory stimulation is provided, the central auditory pathway continues to develop, but it is delayed by the duration of deafness, suggesting a limited form of auditory plasticity. Other studies further suggest that the plasticity of central auditory pathways is maximal only for a restricted period of about 3.5 years in early childhood [10, 12]. If the hearing system is stimulated within that period, the P1 morphology and latency reach age-normal values within 3 to 6 months following the beginning of auditory stimulation. By contrast, if the auditory system does not receive adequate stimulation for more than 7 years, then most children exhibit a delayed P1 latency and an abnormal large P1, even after years of implant use [10, 12].

In the present study, all children with hearing loss experienced a period without any stimulation with hearing aids, since their hearing loss was identified between the age of 20 months and 5 years (Table 1). During this period of deprivation, the maturation of the central auditory nervous system could have been slowed down. The P1 amplitude observed in children with hearing loss could be the reflection of limited plasticity. However, the amplification provided by the hearing aids could have certainly contributed to get under way the maturational processes but it was not probably sufficient to supply entirely the effect of the auditory deprivation.

Two out of four cortical auditory potential components—N1 and P2—were less defined in children with hearing loss compared to their peers with normal hearing. These two components do not emerge consistently until the age of 8 to 11 years in children with normal hearing [13, 24, 26]. The absence of these peaks or their affected morphology in children with hearing loss could be another manifestation of a delayed maturation of the central auditory nervous system. This interpretation is consistent with a study reporting that N1 and P2 are either delayed in developing or absent in children with a cochlear implant [26].

Regarding the N2 maturation in children with normal hearing, N2 amplitude has an initial increase between the age of 5 to 11 years [24] followed by a gradual decline from late childhood to midadolescence [27, 28] and finally N2 amplitude reaches adult values by age 17 [24]. However, there is no general consensus regarding the development of peak latency, with some studies showing a decline [29], no change, [27] or an increase in latency with age [24]. The maturation effect was examined at central (Cz, C3, and C4) and at frontal (Fz) electrodes in 118 subjects [24]. The N2 latency increased significantly as a function of age at central electrodes with no maturational change at the frontal electrode. However, for the children between 9 and 10 years old, the latency values were similar at the four electrode sites [24]. Based on this study [24], the reduction in amplitude and in latency of N2 in children with hearing loss in the present set of data could be explained by a delay in maturation of the central auditory nervous system. Alternately, based on other studies (e.g., [13, 29]), the reduction of N2 latency could be related to a more mature system. However, it seems counter-intuitive that the late component (N2) should mature more rapidly in children with hearing loss than in children with normal hearing. Taking into account the increased P1 amplitude and the abnormal morphology of N1 and P2, the N2 changes would rather militate in favor of delayed maturation in children with hearing loss.

### 4.4. Deficit in the Central Auditory System

The greater amplitude of P1 with a concomitant reduction in N2 amplitude and the less well-defined N1-P2 components could also indicate a deficit in central auditory processing. The anomalies have been reported in central auditory late latency components in children with language-based learning problems (LPs) [30]. Albeit displaying normal hearing sensitivity, these children had abnormalities in neurophysiological encoding marked by different patterns in amplitude or latency compared to their control peers. In fact, one normal category and three atypical categories based on cortical responses of children with LP were found. The atypical category 1 included children with a delayed P1 latency and no evidence of N1 or P2 component. The atypical category 2 was composed of children having normal P1 but delayed N1 and P2 responses. For the atypical category 3, children had generally low-amplitude responses [30]. Although N2 properties were not specifically examined in this study, observations from their results suggest that N2 amplitude and latency values were abnormal (low-amplitude and/or delayed latency) for children in the three atypical categories. These atypical responses might represent a general decrease in synchronous activity, indicating an immature development of the central auditory pathways or slower processing mechanisms [30].

### 4.5. Mismatch Responses

Similar patterns of results were obtained in the two groups of children with the negative mismatch response measured in the 150–200 ms window. These results suggest that the auditory system can discriminate sounds, being the verbal or nonverbal, and that this pattern of discrimination can be found in children with hearing loss as well as in children with normal hearing. They further suggest that the amplification provided by the hearing aids could have contributed to get under way the maturational processes, allowing the children to develop adequate discriminative abilities.

A positive MMR was measured in the 200–300 ms window with the pair of 1 kHz pure tone and wide-band noise only. Results showed that the amplitude of this positive MMR was significantly smaller in children with hearing loss than that observed in children without hearing loss. This result may simply be related to the fact that children with hearing loss have, as stated above, a smaller N2 amplitude in response to the standard stimuli compared to normal hearing children.

The negative MMR was also found to differ according to stimulus type. When the stimuli were simple, (the pair 1 kHz and wide-band noise), the MMR had an earlier latency compared to more complex stimuli, such as the nonverbal and verbal /ba/-/da/. The effect of stimulus type on ERP results has also been reported by other studies [31, 32]. Those and the present results confirm that simple stimuli are more rapidly processed within the central auditory system in comparison to complex stimuli.

## 5. Conclusion and Clinical Implications

Although obtained in a limited number of children and in a restricted age range, these preliminary findings indicate that reduced auditory input early in life has an impact on the development of central auditory functions reflected by the specific patterns of CAEPs. The interaction and the combination of at least two factors, delay in maturation and deficit in the central auditory system, could contribute to the pattern of results obtained in children with hearing loss. The data further indicate that sensory hearing loss affects differently the earlier cortical component P1 compared to the later component N2. Moreover, the findings suggest that CAEPs can be more sensitive markers of the effects of sensory hearing loss than are mismatch responses in children with mild to moderately severe hearing loss. Measuring P1 and N2, as the neurophysiological markers in children with hearing loss, can provide an objective assessment of the maturation of their central auditory system. For well-trained audiologists with CAEPs, results can be easily interpreted. P1 and N2 amplitude measured before and after a given auditory training program may reflect the efficiency of the program and confirm the plasticity of the auditory pathways. Also, with these two neurophysiological components, audiologists may determine whether appropriate stimulation is being provided by a hearing aid or cochlear implant, and based on the findings, they may adjust the auditory training program. However, the CAEPs measures should be adapted before being implanted as an assessment tool in clinics and its cost effectiveness has to be assessed. In the near future, studies will take into account the clinical testing conditions by reducing the number of recording channels (limited to frontal sites) in order to be suitable to clinical equipments and also by developing normative data.

## Figures and Tables

**Table 1:** Data of nine-to ten-year-old children with hearing loss: age (years; months); age of hearing aids fitting (H/A); sex and hearing loss measured in the right ear at 250 to 8000 Hz (NT: not tested); and stimulus presentation level (dB HL).

Participant	Age	H/A	Sex	Hearing threshold (dB HL)						
				250	500	1000	2000	4000	8000	Presentation level (HL)
1	9;07	3;00	F	35	50	60	60	60	NT	90
2	9;08	5;00	F	30	30	65	65	30	15	85
3	10;04	1;08	F	80	100	100	95	85	75	105
4	9;10	5;00	F	30	40	50	60	50	NT	85
5	9;11	4;00	F	40	40	45	50	45	30	85
